# Association between deletions in the preS1/2 region of the hepatitis B virus genome and persistently abnormal ALT levels in patients with chronic hepatitis B treated with nucleos(t)ide analogs

**DOI:** 10.1007/s00705-025-06480-6

**Published:** 2025-12-18

**Authors:** Yurina Sato, Kotaro Doi, Jun Inoue, Masashi Ninomiya, Mio Tsuruoka, Kosuke Sato, Masazumi Onuki, Satoko Sawahashi, Keishi Ouchi, Kengo Watanabe, Hirofumi Niitsuma, Atsushi Masamune

**Affiliations:** 1 Tohoku University School of Medicine, Sendai, Japan; 2 Division of Gastroenterology, Tohoku University Graduate School of Medicine, 980-8574 Sendai, Japan; 3 Institute for Excellence in Higher Education, Tohoku University, Sendai, Japan

## Abstract

Patients with chronic hepatitis B (CHB) are at risk of developing hepatocellular carcinoma (HCC) during treatment with nucleos(t)ide analogs (NAs). Several factors, including abnormal ALT levels during NA treatment, are associated with HCC risk; however, the mechanisms of ALT abnormalities remain unknown. Here, deletions in the preS1/2 region of the hepatitis B virus (HBV) genome were evaluated in the context of persistent ALT abnormalities after NA treatment. Out of the 198 patients with CHB who had been treated with NAs, 40 who had been treated with entecavir, tenofovir disoproxil fumarate, or tenofovir alafenamide fumarate as the first-line therapy and from whom serum samples had been collected before NA treatment were selected for analysis of the infecting virus. Total DNA was extracted from the samples and subjected to nested PCR to detect deletions in the preS1/2 region of the HBV genome. Patients for whom deletions were found in ≥ 40% of the amplicons were classified as the "high-del" group, and the others were the "low-del" group. The preS1/2 region was amplified from 39 (97.5%) of the 40 samples, and a deletion was found in 19 of these patients (48.7%). The cumulative incidence of HCC was found to be significantly higher in the high-del group than in the low-del group (25% vs. 7% at 2 years, *P* = 0.021). The high-del group also had significantly higher ALT levels than the low-del group after 1 year of NA treatment (35 vs. 26 U/L, *P* = 0.020). In summary, patients with CHB with frequent preS1/2 deletions showed higher ALT levels after NA treatment and a higher risk of developing HCC.

**Supplementary Information:**

The online version contains supplementary material available at 10.1007/s00705-025-06480-6.

## Introduction

Chronic hepatitis B virus (HBV) infection is a leading cause of liver cirrhosis and hepatocellular carcinoma (HCC), accounting for the majority of primary liver cancers [1]. Globally, approximately 257.5 million people were estimated to be chronically infected with HBV in 2022 [2], and the number of cases of HBV-related primary liver cancer was estimated to be 288,110 in 2021 [3]. Although the incidence of HBV-related liver disease decreased between 2000 and 2021, in over one-third of the countries worldwide, the incidence of HBV-related primary liver cancer increased [3]. Patients with chronic hepatitis B (CHB) are treated with nucleos(t)ide analogs (NAs) because of their efficacy and low incidence of side effects [4–6]. However, many patients require long-term treatment, as NA treatment rarely achieves a functional cure for HBV infection [7].

Clinically, the estimation of HCC risk in patients with CHB is challenging, and several factors and risk scores associated with the risk of HCC have been reported [8, 9]. We previously reported that abnormal alanine aminotransferase (ALT, ≥ 31 U/L) levels at 1 year of NA treatment were associated with HCC development [10]. In addition, we developed a fibrosis and ALT-1 (FAL-1) score based on the FIB-4 index and ALT levels at 1 year of NA treatment to estimate the risk of HCC [11, 12]. However, the reasons for persistent ALT abnormalities during treatment without detectable serum HBV DNA have not been sufficiently clarified [13, 14].

HBV has a 3.2-kb, circular, partially double-stranded DNA genome with four partially overlapping open reading frames, including the preS/S gene, which encodes the large, middle, and small hepatitis B surface proteins (LHBs, MHBs, and SHBs) [15]. The LHBs consists of the preS1, preS2, and S domains, and the MHBs consists of the preS2 and S domains [15]. Deletions in the preS1/2 region have been shown to be associated with the progression of liver fibrosis and the development of HCC [16–18]; moreover, a preS2 deletion mutant has been shown to cause mitochondrial dysfunction and endoplasmic reticulum (ER) stress, resulting in the development of HCC [19, 20]. Such cellular damage may cause persistent ALT elevation, but the actual association remains unclear. This study focused on the association between preS1/2 deletions and ALT abnormalities during NA treatment.

## Materials and methods

### Patients

**Fig. 1:**
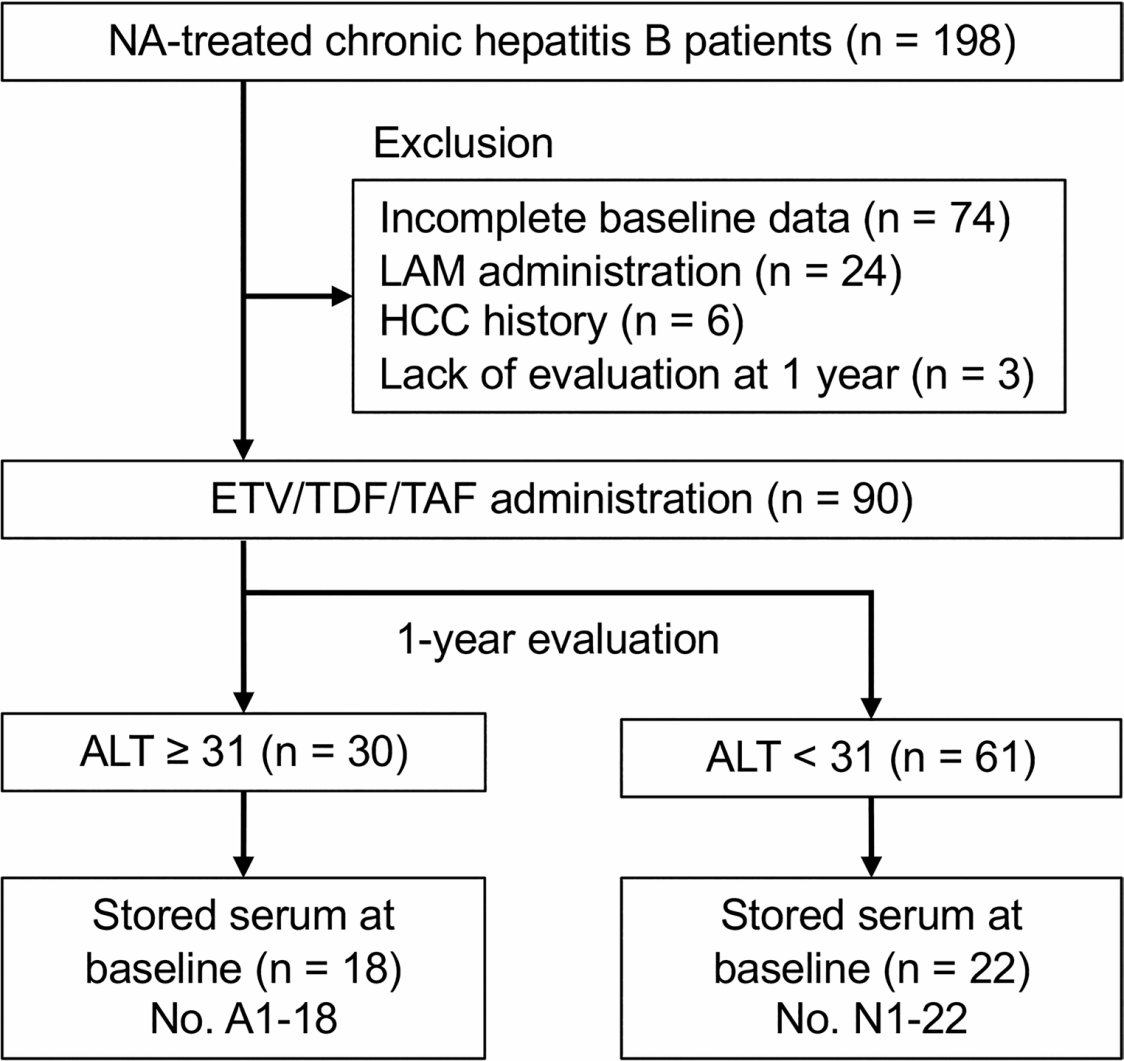
Flow of patients in this study. ALT, alanine aminotransferase; ETV, entecavir; LAM, lamivudine; NA, nucleos(t)ide analog; TAF, tenofovir alafenamide fumarate; TDF, tenofovir disoproxil fumarate

A flowchart of patient inclusion is shown in Fig. 1. Among the 198 patients with CHB treated with NAs, 74 with incomplete baseline data, 24 treated with lamivudine (LAM), six with a history of HCC, and three without follow-up evaluation after 1 year of NA treatment were excluded. Among the remaining 90 patients who were treated with entecavir (ETV), tenofovir disoproxil fumarate (TDF), or tenofovir alafenamide fumarate (TAF) as the first-line NA, 40 whose serum samples were collected and stored at -30℃ before NA treatment were evaluated. Written consent was obtained from all of the patients. The FIB-4 index was calculated as follows: FIB-4 = age (years) × aspartate aminotransferase (AST, U/L)/[platelet, 10^3^/µL) × √alanine aminotransferase (ALT, U/L)] [21]. The FAL-1 score was calculated using FIB-4 index and ALT levels at 1 year after initiation of NA treatment. Using an applicable number of FIB-4 ≥ 1.58 and ALT levels ≥ 31, the score was determined as 0, 1, or 2 [11].

### Amplification of the preS1/2 region of the HBV genome

Total DNA was extracted from stored serum samples and subjected to nested PCR to detect deletions in the preS1/2 region of the HBV genome. The PCR products were separated by agarose gel electrophoresis, and amplicons with deletions were quantified by image analysis. Samples with amplicons shorter than 221 bp were considered to contain an HBV genome with deletion mutations in the preS1/2 region. Detailed information is provided in Supporting Information.

### Identification of the deleted region

PCR products shorter than 221 bp were excised from an agarose gel and purified using a QIAquick Gel Extraction Kit (QIAGEN GmbH, Hilden, Germany). The sequences of the purified products were determined by the Sanger method, using a BigDye Terminator v3.1 Cycle Sequencing Kit (Thermo Fisher Scientific, Waltham, MA) and a 3500xL Genetic Analyzer (Thermo Fisher Scientific). The sequences were analyzed using Genetyx-Mac (ver.18.0.4, Genetyx Corp., Tokyo, Japan). The deleted regions were identified by alignment with the previously reported sequence of HBV genotype C (HBV/C) (GenBank/EMBL/DDBJ accession no. AB033550).

### Statistical analysis

Statistical analysis was performed using the chi-squared test to compare proportions between two groups, and the Wilcoxon rank-sum test was used to compare continuous variables between groups. Cumulative incidences were estimated using the Kaplan−Meier method and compared using the log-rank test. Differences with *P*-values < 0.05 were considered statistically significant. All statistical analysis was performed using JMP version 16.2.0 (SAS Institute Inc., Cary, NC, USA). Data were visualized using GraphPad Prism version 9.5.1 (GraphPad Software Inc., La Jolla, CA, USA).

## Results

### Clinical characteristics of patients

The clinical characteristics of the 40 patients eligible for inclusion in this study are presented in Table 1. The median patient age was 51 years, and 67.5% of the patients were male. The median serum HBV DNA level was 7.3 log IU/mL, and 48.6% of the patients were positive for the hepatitis B e antigen (HBeAg). One patient was infected with HBV genotype A, nine with genotype B, and 29 with genotype C. Six of the 40 patients (15.0%) developed HCC during a median follow-up of 96 months. Hepatic steatosis was observed in 32.4% of the patients who did not develop HCC but was not observed in any of the six HCC patients (*P* = 0.039). HBV/C was more prevalent in patients who developed HCC than in those who did not (100% vs. 70.6%, respectively), but this difference was not statistically significant.

**Table 1:** Clinical characteristics according to HCC development

Factor	Total	HCC (-)	HCC (+)	*P*-value^a^
	n = 40	n = 34	n = 6	
Age (years)	51 (43–59)	49 (42–58)	58 (848 − 65)	0.129
Sex (male/female)	27/13	22/12	5/1	0.345
Body mass index (kg/m^2^)	23.3 (21.4–25.8)	22.8 (21.2–25.3)	25.1 (23.2–27.9)	0.155
HBV genotype (non C/C)	10/29	10/24	0/5	0.073
HBeAg (+/-)	17/18	14/15	3/3	0.939
HBV DNA (log IU/mL)	7.3 (6.5–8.3)	7.2 (6.4–8.3)	7.4 (6.9–8.5)	0.383
HBsAg (IU/mL)	3686 (611–9576)	5509 (617-10290)	858 (571–1391)	0.169
Total bilirubin (mg/dL)	0.9 (0.7–1.2)	1.0 (0.7–1.2)	0.8 (0.7–0.9)	0.322
AST (U/L)	55 (34–136)	59 (33–119)	44 (32–197)	0.691
ALT (U/L)	72 (38–222)	74 (41–228)	43 (32–246)	0.315
Albumin (g/dL)	4.0 (3.6–4.3)	4.1 (3.7–4.3)	4.0 (3.5–4.2)	0.424
Platelets (×10^4^/µL)	16.3 (12.8–20.8)	16.5 (13.5–20.3)	14.8 (11.0-24.5)	0.762
Alfa-fetoprotein (ng/mL)	6.8 (3.6–16.3)	5.6 (3.4–25.6)	8.0 (5.6–13.5)	0.584
FIB-4 index	2.46 (1.56–3.58)	2.28 (1.51–3.50)	3.15 (2.22-4.00)	0.316
Hepatic steatosis (+/-)	11/29	11/23	0/6	**0.039**
NA (ETV/TDF/TAF)	28/5/7	22/5/7	6/0/0	0.094
Observation period (months)	96 (51–154)	102 (57–147)	96 (48–183)	0.762

Data are shown as the median (interquartile range) or number

^a^HCC (-) vs. HCC (+). *ALT* alanine aminotransferase, *AST* aspartate aminotransferase, *ETV* entecavir, *FIB-4*, fibrosis-4, *HBeAg* hepatitis B e antigen, *HBsAg* hepatitis B surface antigen, *HBV* hepatitis B virus, *HCC* hepatocellular carcinoma, *NA* nucleos(t)ide analogue, *TAF*, tenofovir alafenamide fumarate, *TDF* tenofovir disoproxil fumarate

### Detection of preS1/2 deletions

**Fig. 2:**
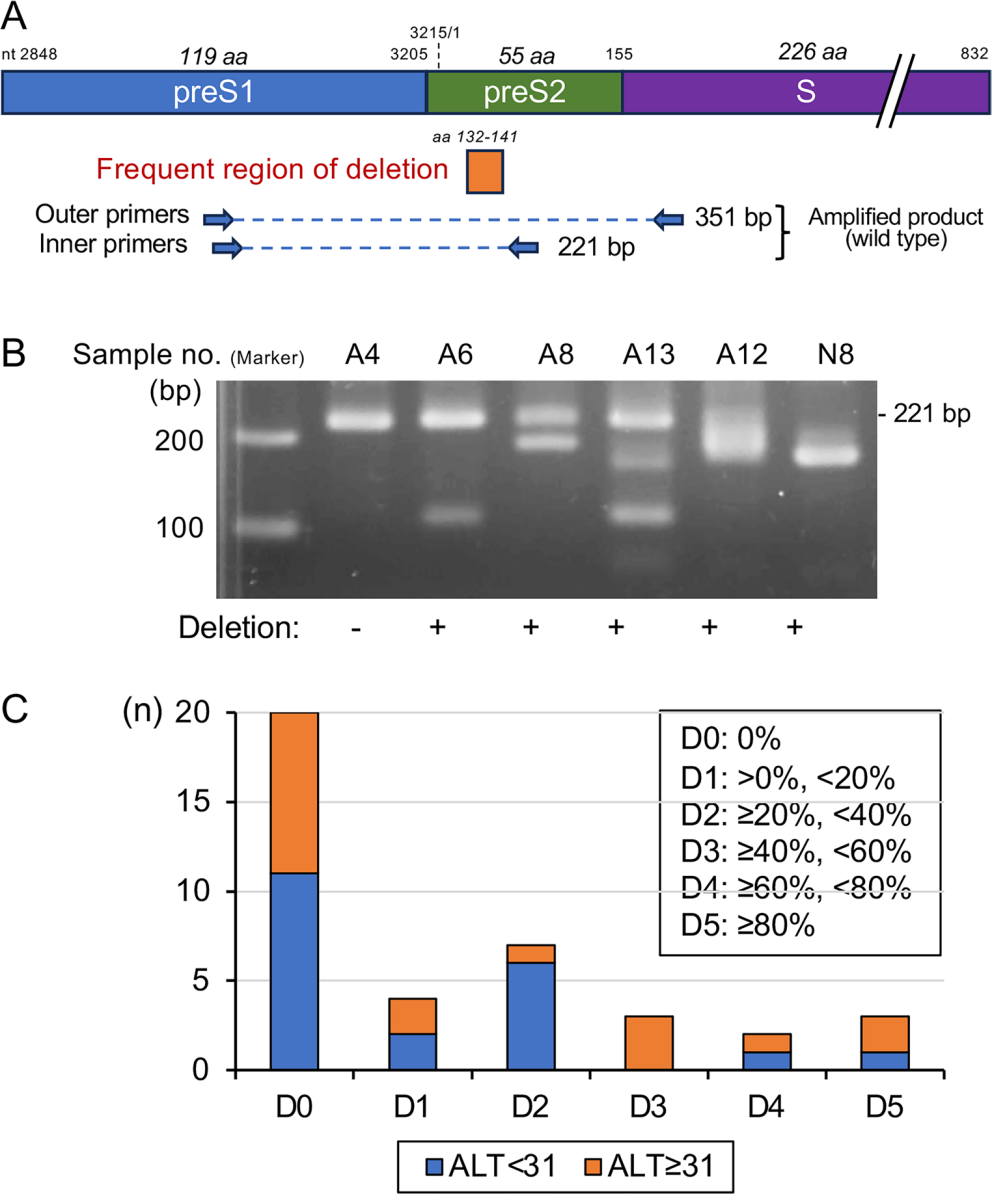
Detection of deletions in the preS1/2 region. (**A**) Schematic diagram of the nested PCR amplification of the preS1/2 region. (**B**) Representative results of the electrophoresis of amplified DNA products. (**C**) The proportion of deletion mutants in each sample was quantified, and the patients were grouped accordingly into D0, D1, D2, D3, D4, and D5. The number of patients in each group according to ALT levels is shown.

The preS1/2 region was amplified by nested PCR from 40 serum samples collected before the start of NA treatment, and 39 samples (97.5%) yielded positive results. Among the 39 positive samples, amplicons shorter than 221 bp were obtained from 19 (48.7%), indicating the presence of deletion mutants in the sample. Examples of the band patterns are shown in Fig. 2B. Using electrophoresis gel images, the signal intensity of each band ≤ 221 bp was quantified, and the proportion of deletion mutants was calculated. Patients were categorized into groups based on the proportion of preS deletion mutants in the sample (D1, 0–20%; D2, 20–40%; D3, 40–60%; D4, 60–80%; D5, ≥ 80%). Patients without deletion signals were classified as D0. Four, seven, three, two, and three patients were grouped into D1, D2, D3, D4, and D5, respectively (Fig. 2C).

**Fig. 3:**
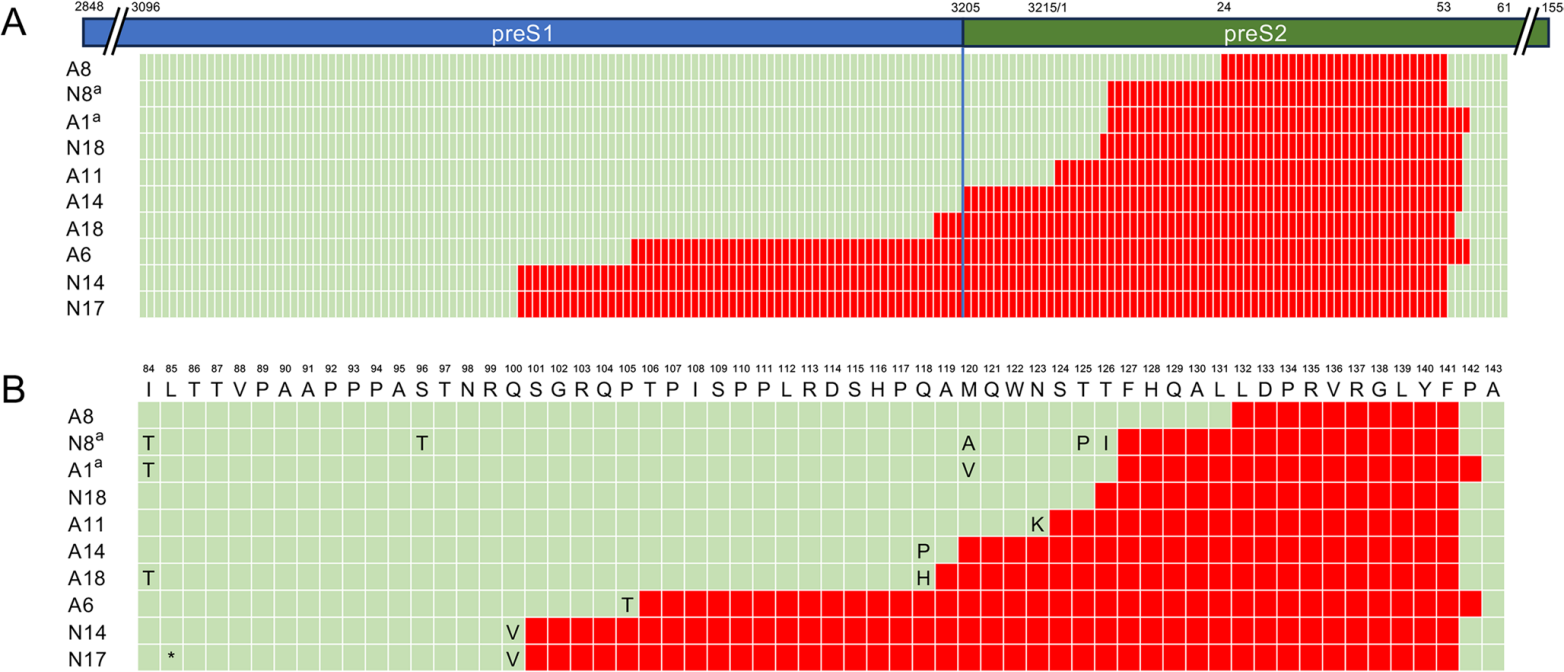
Identification of deletions in the preS1/2 region by Sanger sequencing. (**A**) Nucleotide deletions in the viral genomes from 10 patients. Red areas indicate deletions. (**B**) Amino acid deletions. The amino acid sequence of HBV/C (accession no. AB033550) is shown at the top. Deleted regions are shown in red, and amino acid substitutions are also shown. ‘*’ indicates a stop codon. ^a^Patients who developed HCC during follow-up after NA treatment

After electrophoresis, bands < 221 bp were excised, and deleted regions were identified using Sanger DNA sequencing. The deleted regions were successfully identified in 10 samples, and in each case the deleted region included nt 24–53 in preS2 (Fig. 3A), corresponding to aa 132–141 (Fig. 3B). Most of the deleted regions contained a previously reported CD8^+^ T-cell epitope, aa 131–139 [22]. Furthermore, two patients (N8 and A1) who developed HCC after NA treatment were found to have viruses with mutations in the initiation codon of preS2 (Fig. 3B).

### Association between preS deletion and HCC development

**Fig. 4:**
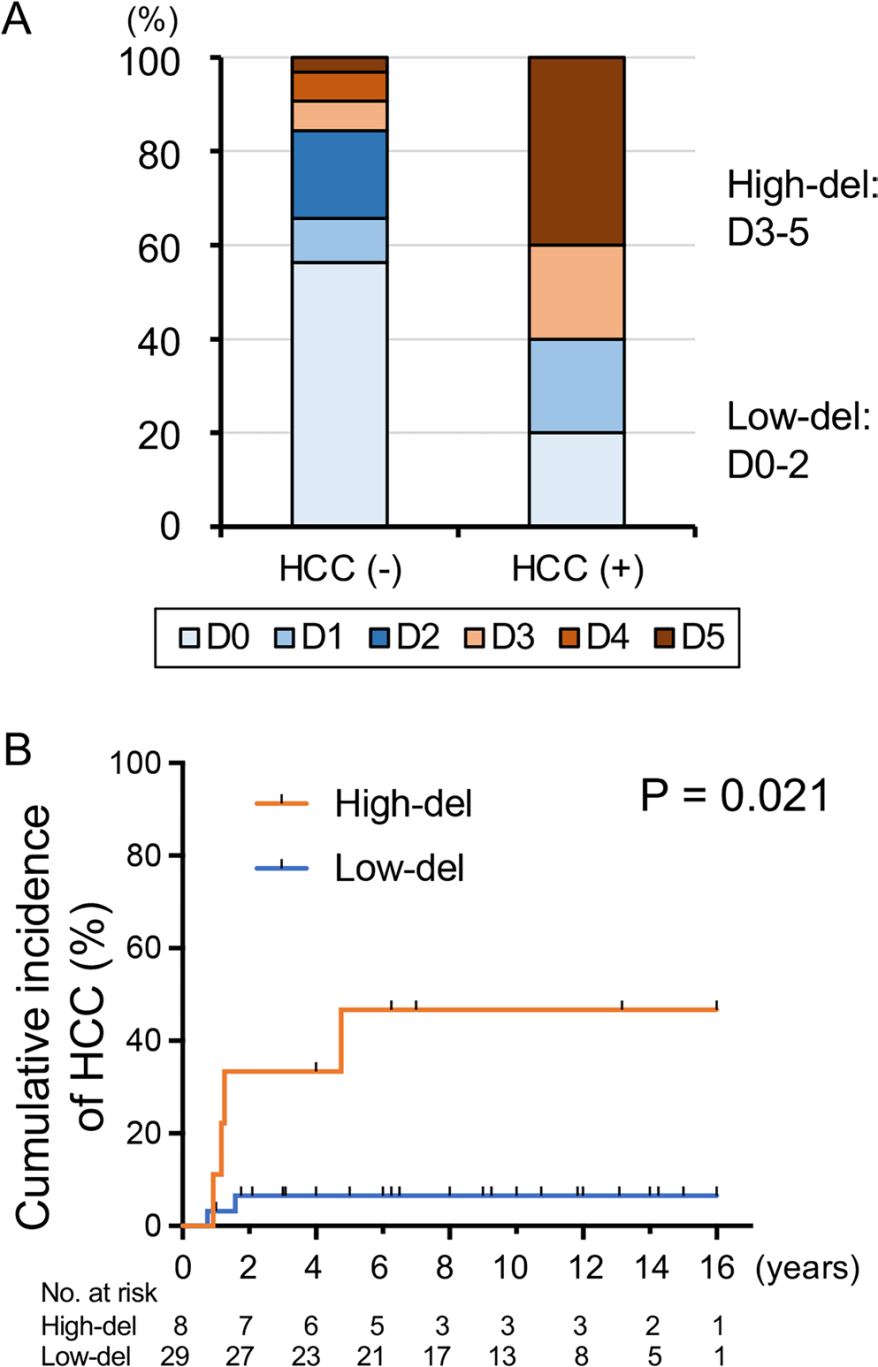
Association between preS1/2 deletion frequencies and HCC development. (**A**) Comparison of D0-D5 group proportions between patients with and without HCC development during the observation period. (**B**) Kaplan−Meier analysis of the cumulative incidence of HCC in the high-del (D3, D4, and D5) and low-del (D0, D1, and D2) groups

To investigate whether the deletion frequency was related to HCC risk, we next compared the distribution of deletion groups between patients with and without HCC, which showed a higher proportion of sequences with deletions in samples from patients who developed HCC (Fig. 4A). When a receiver operating characteristic analysis was performed for preS deletion (%) vs. HCC development, the area under the curve was 0.744, and the cutoff for preS deletion was 43.9% (Supplementary Fig. S1). Based on this internal analysis, we defined patients with a deletion signal ≥ 40% of the total amplified signal (corresponding to D3, D4, and D5) as the "high-del" group and those with < 40% (D0, D1, and D2) as the "low-del" group. Kaplan−Meier analysis showed that the cumulative incidence of HCC was significantly higher in the high-del group than in the low-del group (*P* = 0.021) (Fig. 4B).

### PreS deletion and persistent ALT abnormality

**Fig. 5:**
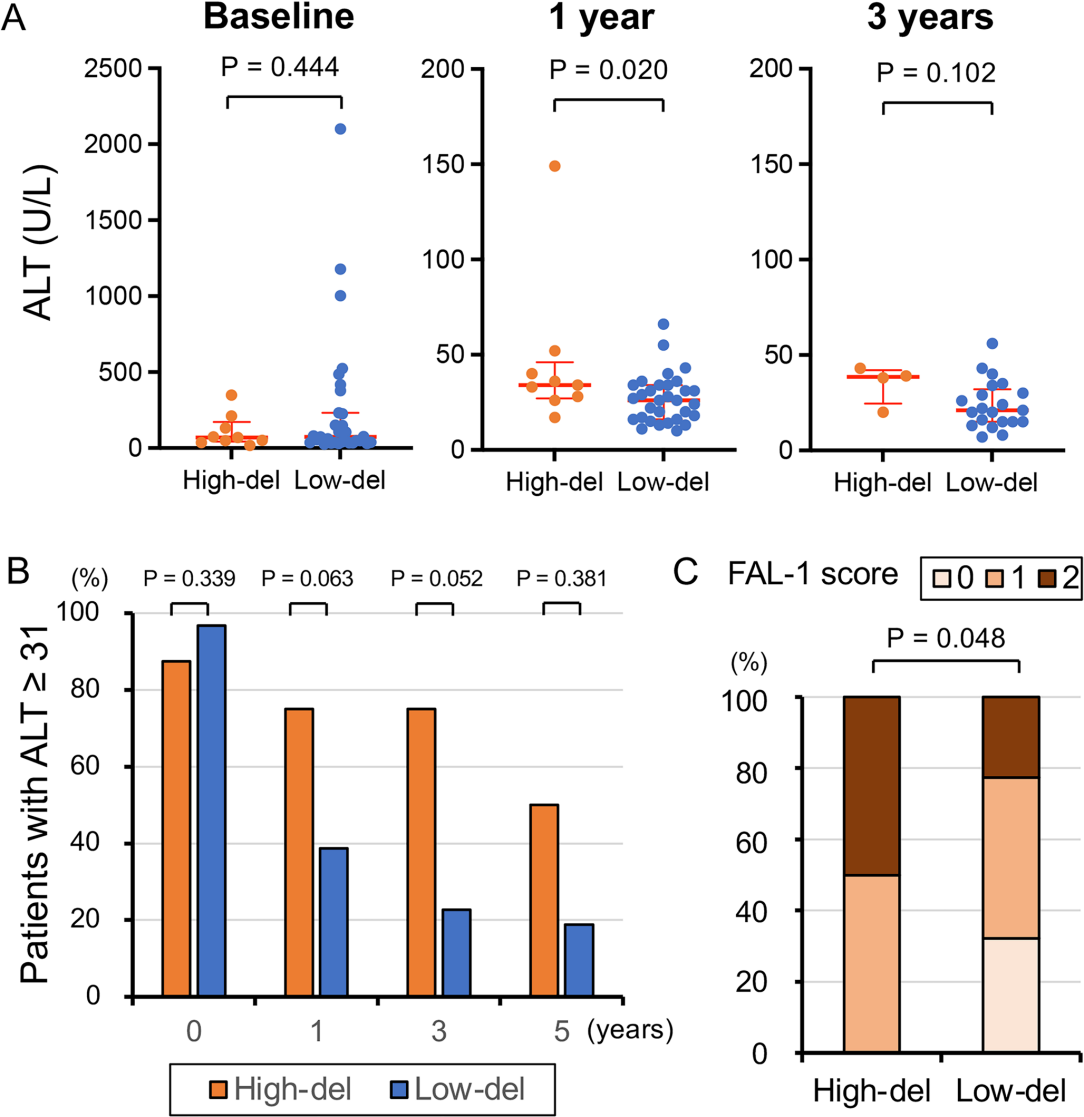
ALT abnormality after NA treatment in the two deletion groups. (**A**) ALT levels were compared between the high-del and low-del groups at baseline, 1 year, and 3 years after the initiation of NA treatment. The red bars indicate the median and interquartile range. (**B**) The proportion of ALT abnormality (≥ 31 U/L) at baseline, 1, 3, and 5 years after the initiation of NA treatment in the high-del and low-del groups. (**C**) The proportion of patients with each FAL-1 score in the high-del and low-del groups

To analyze the association between preS deletion and persistent ALT abnormalities, the ALT levels of the preS deletion groups at baseline and after NA treatment were compared (Fig. 5A). Although there was no significant difference in ALT levels between the high-del and low-del groups at baseline (59.5 vs. 74.0 U/L, *P* = 0.444), the difference was significant after 1 year of NA treatment (35.0 vs. 26.0 U/L, *P* = 0.020). A similar trend was observed at 3 years, but the difference was no longer significant (38.5 vs. 21.0 U/L, *P* = 0.102), probably due to the small sample size. ALT abnormality was defined as ≥ 31 U/L according to previous studies [10, 11]. The proportion of patients with abnormal ALT levels was higher in the high-del group at 1, 3, and 5 years of NA treatment, but these differences were not significant (Fig. 5B). The course of ALT abnormalities from 1 to 5 years in each patient, according to HBV genotype, FAL-1 score, and deletion frequency, is shown in Supplementary Fig. S2. The deletion positions and lengths for each patient are shown in Supplementary Fig. S3. Two patients who developed HCC had relatively short deletions (45–48 nt) in the preS2 region. In contrast, two patients with a long deletion of 123 nt from the preS1 to the preS2 region showed a FAL-1 score of 0, indicating a low risk of HCC.

Finally, FAL-1 scores were compared between the high- and low-del groups. The number of patients with an FAL-1 score of 0, 1, and 2 was 0, 4, and 4, respectively, in the high-del group and 10, 14, and 7, respectively, in the low-del group (*P* = 0.048), indicating that the score was significantly higher in the high-del group (Fig. 5C).

## Discussion

The primary findings of this study were as follows: (1) Deletions in the preS1/2 region were found in samples from 48.6% of patients with CHB, using a PCR-based method. (2) The presence of preS1/2 deletions was associated with HCC development during NA treatment. (3) ALT levels after 1 year of NA treatment were significantly higher in patients with a high frequency of preS1/2 deletions than in those with a low frequency. An association between preS1/2 deletions and HCC development in patients with CHB has been reported previously in a meta-analysis [23], but to the best of our knowledge, this is the first report to demonstrate an association between preS1/2 deletions and ALT abnormalities during NA treatment. Previously, we reported that the cumulative incidence of HCC was significantly higher in patients with ALT abnormality (≥ 31 U/L) [10, 11], but the mechanism was unclear. Because preS2 deletions result in endoplasmic reticulum stress and mitochondrial dysfunction [19], they might be the cause of persistent ALT abnormalities, due to these effects on hepatocytes. While detection of preS2 deletions is uncommon in clinical settings, ALT levels are routinely monitored in the management of patients with CHB. Persistent ALT abnormalities during NA treatment, especially after one year of therapy, might reflect direct hepatocyte damage caused by preS2 deletion mutants. In most cases, ALT levels decrease after the initiation of NA treatment; however, even minor abnormalities should be carefully monitored to assess the risk of HCC. Hepatic steatosis is one of the reasons for ALT abnormality during NA treatment, but it should be noted that the patients who developed HCC showed a significantly lower frequency of hepatic steatosis (Table 1), consistent with previous reports [24, 25]. However, the effects of steatosis on CHB progression are still controversial [26]. One proposed mechanism involves the redistribution of cytoplasmic HBV antigens due to intracellular fat accumulation, which induces hepatocyte apoptosis and consequently suppresses viral replication [27]. Our results suggest that patients undergoing treatment with NA who still have abnormal ALT levels without hepatic steatosis have a risk of HCC due to the presence of preS2 deletions. Although our findings support a possible association between preS2 deletions and ALT abnormalities, this was not confirmed experimentally in this study. Further mechanistic studies are needed to determine whether preS2 deletions are a direct cause of hepatocyte injury.

The frequent occurrence of deletions in the preS2 region is believed to be due to the high variability of the spacer region of the reverse transcriptase gene, which overlaps with the preS2 region in the HBV genome [28]. Additionally, the MHBs protein is not crucial for the life cycle of HBV, unlike LHBs and SHBs, which are important for viral entry, assembly, and secretion [28]. A recent study demonstrated that replacement of nt 17–49 in the preS2 region with an HiBiT tag resulted in a viable virus that was able to complete its life cycle [29]. The regions that are frequently deleted overlap with T cell epitopes [30], suggesting that an accumulation of deletion mutants might occur as a result of immune selection pressure [31].

The mode of HBV transmission can differ among the different genotypes [32], affecting the clinical course of infection [33]. In this study, deletions in the preS1/2 region were detected more frequently in patients infected with HBV/C than in those infected with genotype B (HBV/B). This is consistent with previous findings, including a matched case-control study by Yeung et al., which demonstrated that preS2 deletions were significantly more prevalent in HBV/C infections and were independently associated with HCC development after adjusting for viral load, HBeAg status, and genotype [34]. It is noteworthy that the reported HBV/B strains had more than two amino acid substitutions in the CD8^+^ T cell epitope (aa 131–139), whereas this site in HBV/C strains rarely had substitutions (Supplementary Fig. S4). We speculate that these substitutions in the epitope reduce immune pressure, making deletions in this region less frequent in HBV/B infections. In a previous age- and sex-matched study, we found that CHB patients with HBV/C showed a higher incidence of HCC than those with HBV/B [35]. The higher frequency of the preS1/2 deletion may be one of the mechanisms underlying the carcinogenic potential of HBV/C. Furthermore, the HBV/B from patient N18 in this study had a stop codon in the preS1 region in addition to a preS1/2 deletion. The proportion of deletion mutants in this patient was estimated to be 29%, and the remaining genomes without deletions encoded complete LHBs. The significance of the stop codon is unclear, but we speculate that LHBs with a deletion may not favor replication and/or that the short preS1 protein may play a role in HBV/B infection.

In this study, relatively short deletions confined to the preS2 region appeared to be associated with worse outcomes, as two of the six patients in this group developed HCC, and two others had a FAL-1 score of 2 (Supplementary Fig. S3). Although this finding suggests that shorter deletions may be more harmful than longer ones, the number of such cases was small, and no definitive conclusion can be drawn. Interestingly, patients with longer deletions spanning the preS1 and preS2 regions did not develop HCC during the observation period and had lower FAL-1 scores. Deletions including the preS1 region can result in decreased replication efficiency. Therefore, longer deletions are unlikely to be observed frequently. The affect of the length of preS1/2 deletions on hepatocyte damage needs to be clarified in future studies.

NAs reduce the risk of HCC in patients with CHB, even in those with preS2-mutant HBV [19]. However, the reduction of serum HBsAg levels and intracellular covalently closed circular DNA (cccDNA) achieved by NA treatment is not ideal [15, 35, 36], suggesting that the suppressive effects on envelope proteins with preS deletions may also be insufficient. Therefore, novel therapies that inhibit the accumulation of preS mutants in the ER might be effective for preventing the development of HCC [15].

In our cohort, no significant associations were observed between preS1/2 deletion status and established hepatocellular carcinoma (HCC) risk factors, including HBsAg levels, HBV DNA, FIB-4 index, or HBeAg status (Table 2). This suggests that preS deletions might represent an alternative and independent pathway contributing to hepatocarcinogenesis. This observation is consistent with findings from a large-scale matched case-control study by Yeung et al. [34], which demonstrated that preS2 deletions were independently associated with HCC development, even after adjusting for viral load, HBeAg status, and HBV genotype. Importantly, Yeung et al. reported that preS deletions were particularly prevalent and impactful in younger patients (< 50 years), underscoring their significance in HCC risk assessment beyond conventional markers.

**Table 2:** Comparison of patient characteristics between patients with and without a high frequency of preS1/2 deletion

Factor	High-del	Low-del	*P*-value
	(n = 8)	(n = 31)	
Age (years)	46 (37–63)	52 (45–59)	0.663
Sex (male/female)	7/1	19/12	0.135
Body mass index (kg/m^2^)	24.7 (21.7–27.3)	22.8 (21.4–25.6)	0.351
HBV genotype (non C/C)	0/8	10/21	**0.020**
HBeAg (+/-)	4/2	13/15	0.364
HBV DNA (log IU/mL)	7.6 (7.0-7.8)	6.9 (6.4–8.3)	0.423
HBsAg (IU/mL)	1391 (17-3110)	5534 (686-10474)	0.162
Total bilirubin (mg/dL)	0.8 (0.7–1.2)	0.9 (0.7–1.1)	0.958
AST (U/L)	44 (32–84)	57 (34–144)	0.434
ALT (U/L)	60 (39–118)	74 (37–233)	0.444
Albumin (g/dL)	4.1 (3.3–4.4)	4.0 (3.6–4.3)	0.944
Platelets (×10^4^/µL)	14.6 (10.7–17.1)	16.6 (13.8–21.1)	0.251
Alfa-fetoprotein (ng/mL)	7.9 (5.5–13.6)	5.0 (3.3–22.2)	0.340
FIB-4 index	2.75 (1.11–3.65)	2.43 (1.54–3.61)	0.958
Hepatic steatosis (+/-)	2/6	9/22	0.820
NA (ETV/TDF/TAF)	6/1/1	21/4/6	0.892
Observation period (months)	96 (77–184)	108 (48–144)	0.486

Data are shown as the median (interquartile range) or number. *ALT* alanine aminotransferase, *AST* aspartate aminotransferase, *ETV* entecavir, *FIB-4* fibrosis-4, *HBeAg* hepatitis B e antigen, *HBsAg* hepatitis B surface antigen, *HBV* hepatitis B virus, *HCC* hepatocellular carcinoma, *NA* nucleos(t)ide analogue, *TAF* tenofovir alafenamide fumarate, *TDF* tenofovir disoproxil fumarate

This study had some limitations. First, the number of patients with stored serum samples was small, and only six patients developed HCC during follow-up. This limited the statistical power, especially in subgroup analysis, and may have introduced selection bias. The results should therefore be interpreted with caution and validated using larger independent cohorts. Second, deletion mutants were detected using a PCR-based method, and it is possible that the percentage of deletion mutants may not be accurate. We employed this approach with the aim of developing a simple and practical method suitable for use in clinical research settings. While this method allowed the identification of major deletion variants, it may underestimate the presence of low-abundance mutants. Deep sequencing offers higher sensitivity and could reveal a broader range of deletion variants. Future studies using this approach with larger cohorts would help to validate and expand on our findings. Third, the cutoff value of 40% used to define high-frequency deletions was determined based on ROC analysis. While this threshold showed reasonable ability to distinguish between patients with and without HCC development, it has not been tested in other populations. Future studies are needed to validate its applicability in broader clinical settings.

In conclusion, this is the first study to demonstrate that deletion of preS1/2 is associated with ALT abnormalities during NA treatment. The presence of deletion mutants can contribute to the development of HCC; hence, ALT abnormalities, even at low levels, should be carefully monitored during NA treatment.

## Electronic Supplementary Material

Below is the link to the electronic supplementary material

Supplementary Material 1 (DOCX 19.6 MB)
