# Supplementary material for: Association between deletions in the preS1/2 region of the hepatitis B virus genome and persistently abnormal ALT levels in patients with chronic hepatitis B treated with nucleos(t)ide analogs
Source: Arch Virol. 2025 Dec 18;171(1):27. doi: 10.1007/s00705-025-06480-6 (PMC12715050; doi:10.1007/s00705-025-06480-6)
Supplement: Supplementary file 1 — Supplementary Material 1 (DOCX 19.6 MB) [file 705_2025_6480_MOESM1_ESM.docx]

**Association between deletion in the preS1/2 region of HBV genome and persistently abnormal ALT in chronic hepatitis B patients who were treated with nucleos(t)ide analogues**

Yurina Sato, Kotaro Doi, Jun Inoue, Masashi Ninomiya, Mio Tsuruoka, Kosuke Sato, Masazumi Onuki, Satoko Sawahashi, Keishi Ouchi, Kengo Watanabe, Hirofumi Niitsuma, Atsushi Masamune

**Supporting information**

**Methods**

**Amplification of the preS1/2 region of the HBV genome**

Total DNA was extracted from stored serum samples using a QIAamp DNA Blood Mini Kit (QIAGEN GmbH, Hilden, Germany) and subjected to nested PCR to detect deletions in the preS1/2 region using PrimeSTAR HS DNA polymerase (TaKaRa Bio Inc., Shiga, Japan). The primers B062 (5′-TTG GGG TGG AGC CCT CAG GC-3′) and B065 (5′-CGC CTG TAA CAC GAG CAG GG-3′) were used in the first round of PCR, and the primers B063 (5′-TGG AGC CCT CAG GCT CAG GG-3′) and B064 (5′-GTT CCK GAA CTG GAG CCA CC-3′) were used in the second round. The primers were placed across a frequently deleted region (aa 132-141) in preS2, which was previously reported [1]. For the wild-type sequence, the 351-bp and 221-bp products were amplified in the first and second rounds of PCR, respectively. The primer positions are shown in Fig. 2A. The amplified products of the second round of PCR were electrophoresed on 3% NuSieve 3:1 agarose gel (Lonza, Basel, Switzerland). Samples with bands shorter than 221 bp were considered to contain an HBV genome with deletion mutations in the preS1/2 region. Images of the gel were analyzed using ImageJ software (National Institutes of Health, Bethesda, MD, USA), and the signal intensity of each band was quantified.

**Reference**

1. Suzuki Y, Maekawa S, Komatsu N, Sato M, Tatsumi A, Miura M, Matsuda S, Muraoka M, Nakakuki N, Amemiya F, Takano S, Fukasawa M, Nakayama Y, Yamaguchi T, Inoue T, Sato T, Sakamoto M, Yamashita A, Moriishi K, Enomoto N (2019) HBV preS deletion mapping using deep sequencing demonstrates a unique association with viral markers. PLoS One 14:e0212559. <https://doi.org/10.1371/journal.pone.0212559>


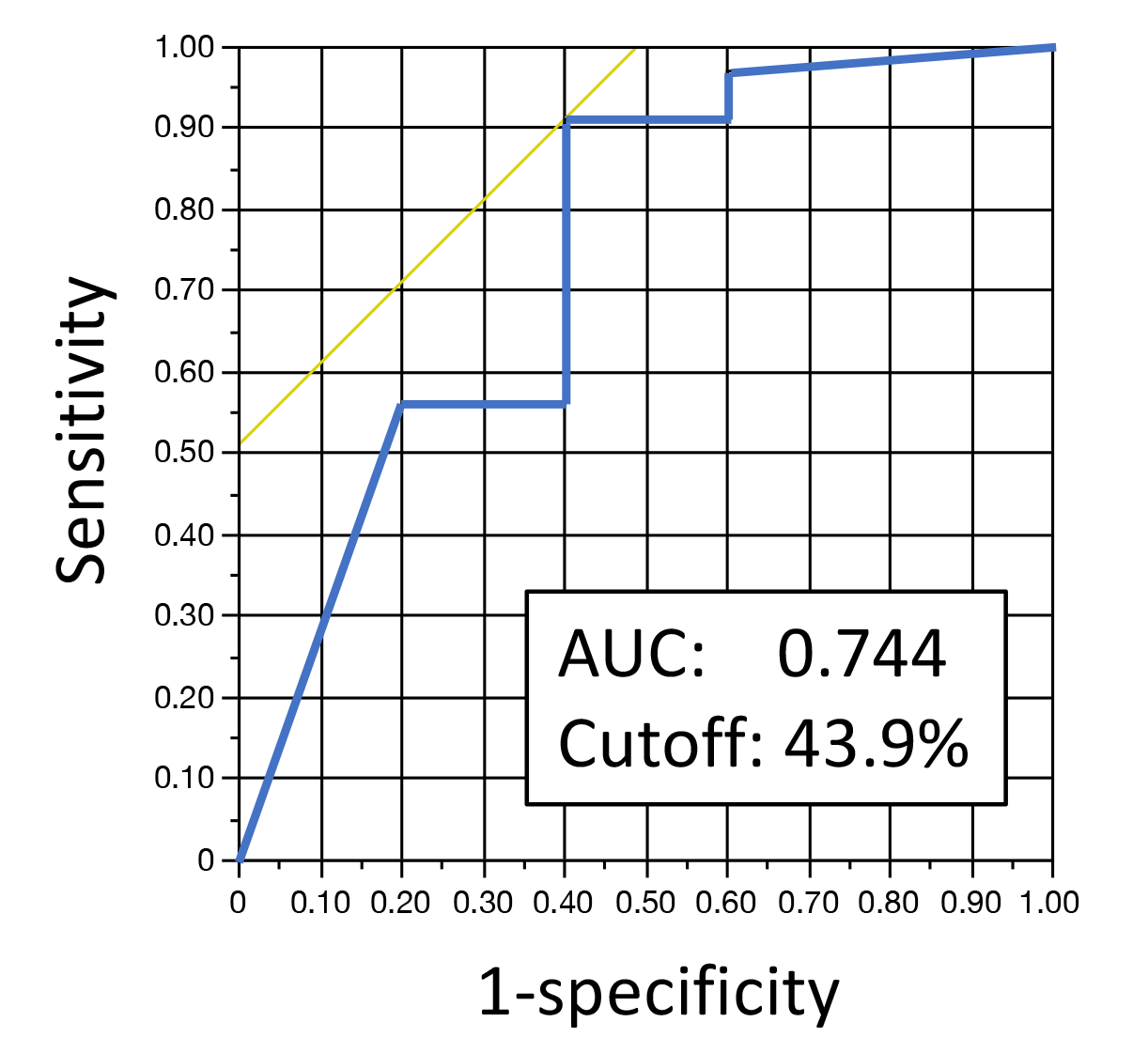
**Fig. S1** Result of receiver operating characteristic analysis for preS deletion (%) and HCC development.


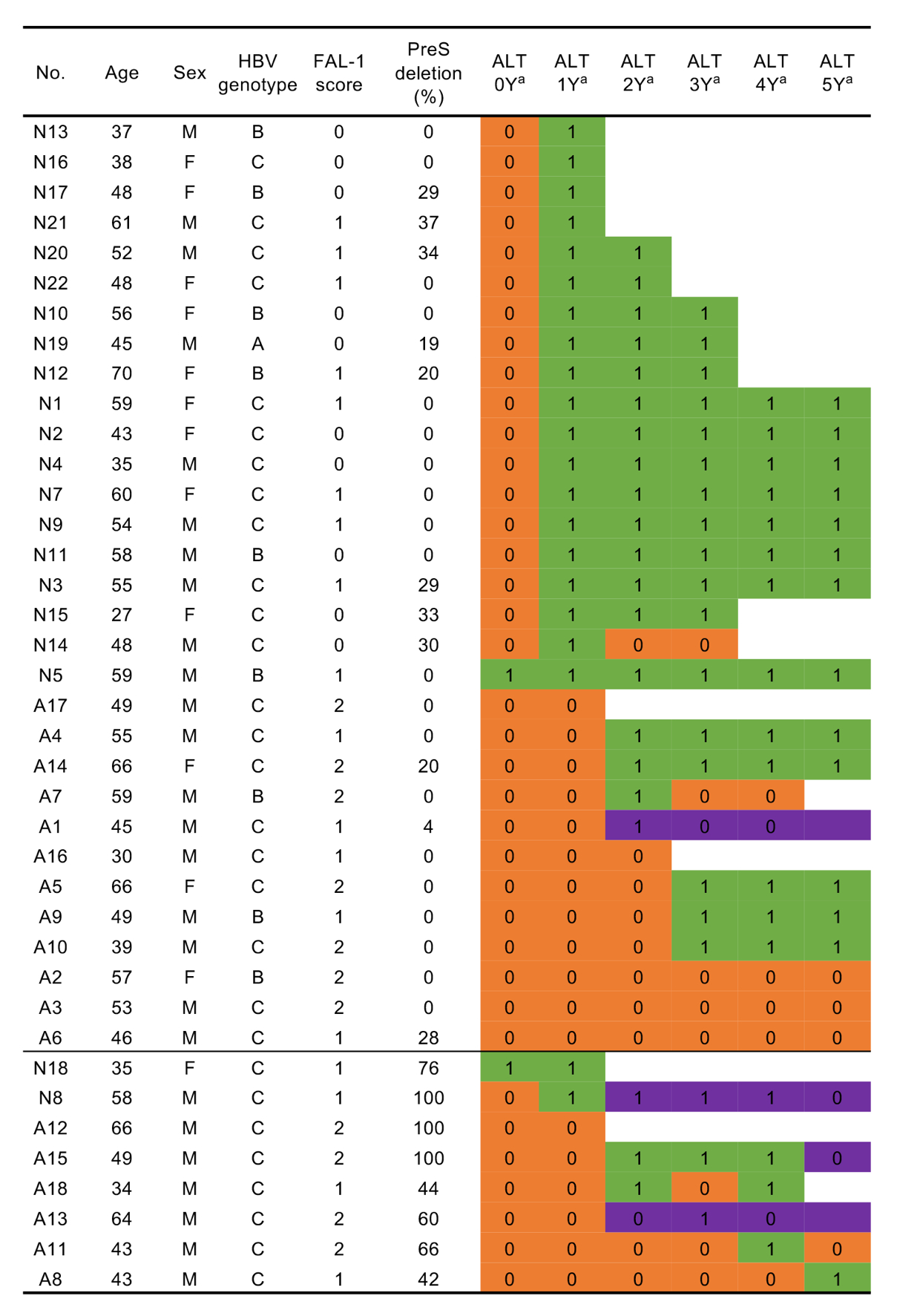
**Fig. S2** List of patients with the course of ALT abnormality from 1 to 5 years. ^a^ ‘0’ indicates abnormal ALT (≥31 U/l), and ‘1’ indicates normal ALT (<31 U/l). Purple areas indicate data after the diagnosis of HCC.


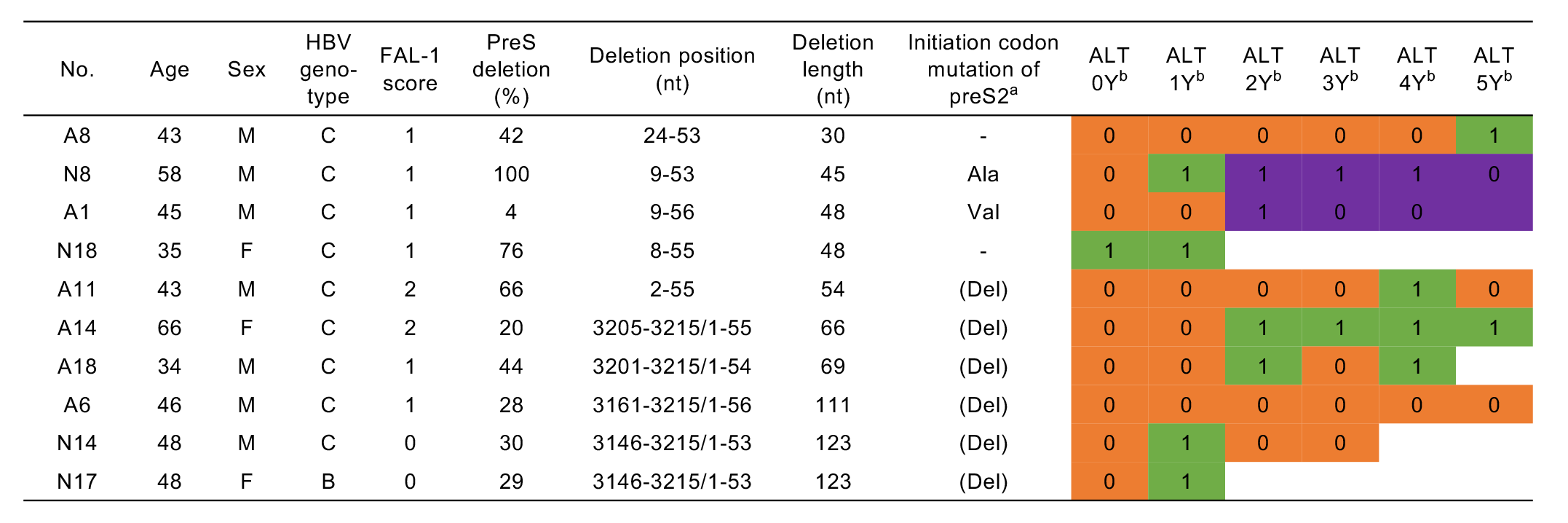
**Fig. S3** Deletion positions and lengths in each patient. ^a^‘-’ indicates no mutation, and ‘(Del)’ indicates that the initiation codon was included in the deletion region. ^b^’0’ indicates abnormal ALT (≥31 U/l), and ‘1’ indicates normal ALT (<31 U/l). Purple areas indicate data after the diagnosis of HCC.


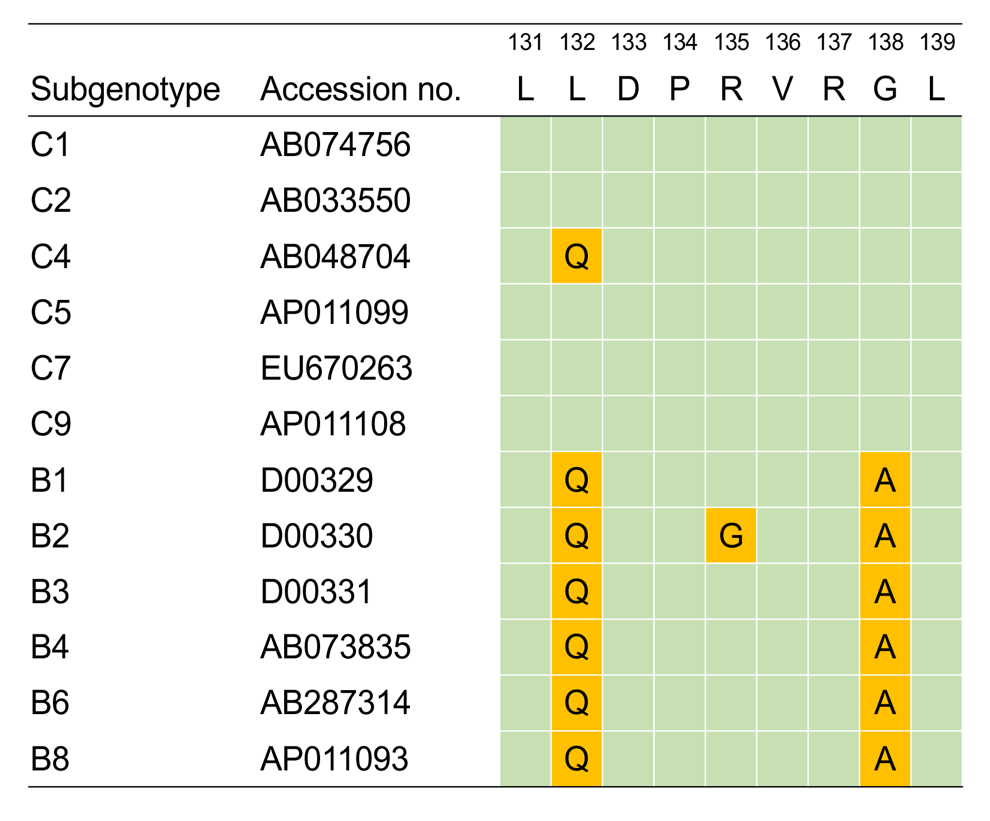
**Fig. S4** Amino acid substitution of the reported T cell epitope in the preS2 region. Results for each 12 representative strains of HBV/C and HBV/B are shown.
